# Annual Meeting of the President and General Council

**Published:** 1920-05-08

**Authors:** 


### KING EDWARD'S HOSPITAL FUND FOR LONDON.

### Annual Meeting of the President and General Council.

The annual meeting of the President and General Council of King Edward's Hospital Fund for London, to receive the Accounts and the Report for the year 1919, was held at St. James's Palace on April 30, the Earl of Donoughmore, K.P., being in the chair.

Those present were: The Governor of the Bank of England (Mr. M. C. Norman), The Rev. A. E. Garvie, D.D., The Rev. W. L. Watkinson, D.D., The Viscount Mersey, The Viscount Farquhar, The Viscount Burnham, Lord Revelstoke, Lord Somerleyton (Honorary Secretary), Lord Stuart of Wortley, The Hon. Sir Arthur Stanley, The President of the Hospital Sunday and Hospital Saturday Funds (The Rt. Hon. The Lord Mayor), The Chairman of the County Council (Mr. John W. Gilbert), The President of the Royal College of Physicians (Sir Norman Moore, Bart.), The President of the Royal College of Surgeons (Sir George Makins), The Rt. Hon. Sir T. Vezey Strong, The Rt. Hon. Sir Horace Marshall, Sir William Church, Bart., Sir Walter Lawrence, Bart., Sir Francis H. Champneys, Bart., Sir William H. Bennett, Sir William J. Collins, Sir Frederick M. Fry (Honorary Secretary), Sir John Craggs, Sir John Tweedy, Mr. John G. Griffiths (Honorary Secretary), and Mr. Yvon R. Eccles.

### The First Resolution.

**Lord Somerleyton** (Hon. Secretary): Lord Donoughmore, my Lords and Gentlemen,—We have only just received the news to-day, or late last night, of the death of Sir Henry Burdett, who was in many ways the originator of the idea of this Fund. As one of its oldest members, I should like to move a resolution expressing our deepest regret at the loss we have sustained and our sympathy with the relatives. Sir Henry Burdett never missed a meeting of the Council since the foundation of the Fund twenty-three years ago. He was also the originator of the solution of that problem which is being discussed so much at the present day, the Uniform System of Hospital Accounts; although Mr. Griffiths and others have had a great deal to do with the working of it, the original idea came from Sir Henry Burdett. I am sure, my Lord, that I am only voicing the general feeling of this Council in expressing our sincere and deep regret.

**Sir William Collins:** My Lords and Gentlemen,—As an Honorary Secretary of the League of Mercy, of which Sir Henry Burdett was for many years the Honorary Treasurer, I wish to associate myself and also the League with this resolution of regret and condolence.

The resolution was put from the Chair, the Council rising in their places, and carried unanimously.

### Change in the Form of the Accounts.

**Lord Revelstoke** (the Honorary Treasurer), in presenting the account of receipts and expenditure and the balance sheet for the year ended December 31, 1919, said that there had been one change in the form of the accounts. As the Council were aware, the Act of Incorporation of the Fund gave rather exceptional powers of investment outside securities authorised by the Trustee Acts, whenever the donors so desired, and ever since the Act came into operation the Funds which must be invested in Trust securities had been shown separately from the Funds to which these special powers applied. These two Funds, formerly designated Trust Fund and Non-Trust Fund, were now called "A" Fund and "B" Fund respectively.

The Council would remember that at the meeting held in December last His Royal Highness the President called attention to the fact that the amount distributed was being increased from £200,000 to £230,000, mainly for the purpose of assisting the hospitals to meet the cost of renewals and repairs deferred owing to the War, and that in order to make this exceptional increase it was estimated that about £50,000 would have to be withdrawn from the reserves created by exceptional legacies and donations received during the War. The Council would notice from the accounts now before them that the amount actually drawn from the reserves was £46,000, or about £4,000 less than was anticipated.

**Lord Donoughmore** formally moved the adoption of the accounts.

The resolution, having been seconded by Sir T. Vezey Strong, was then put and carried unanimously.

**Sir Frederick M. Fry** (Honorary Secretary) presented the draft report of the Council for the year 1919 (see p. 138).

### The President's Powers Committee.

**The Earl of Donoughmore** in moving the adoption of the report, said:—

My Lords and Gentlemen,—I have first of all the honour of reading the following cablegram from His Royal Highness the Prince of Wales, President of the Fund:—

> I much regret that my absence in Australasia makes it impossible for me to be present at the annual meeting this year. Please convey to the General Council my best wishes for the continued success of the Fund.
>
> Edward, P.

I am sure we shall all desire to convey to His Royal Highness our loyal thanks for his message and our sincere wish that in his journey this year he will repeat the triumphant success which he undoubtedly achieved on his tour last year. (Hear, hear.)

I do not think I need say very much in commending the Report to you, especially as it deals with matters of which you have a much more intimate knowledge than I have myself. I should like first of all to say that one of our colleagues on the President's Powers Committee, Lord Finlay, has asked me to express his great regret that his engagements prevent his being here to-day; I am sure we can all understand his absence, as he is a very busy man. We are deeply sensible of the privilege His Royal Highness has conferred upon us in delegating to us, during his absence his powers as President under the Act of Parliament incorporating the Fund. The President being merely absent for a time from the United Kingdom, and not wholly out of reach in case of urgency, the exercise of these powers, which, as you know, are very extensive, does not involve the same degree of responsibility as was undertaken by the three Governors who did the work so well from 1910 to 1919, when the office of President was in abeyance.

Nevertheless, as everyone must realise, the responsibility exercised jointly by the President's Powers Committee and the General Council at the present moment is by no means a light one. We all confidently expect great things from the appointment of the Prince of Wales as President, and we have good reason for expecting them, knowing the records of his predecessors in the Presidency, his late Majesty King Edward and his present Majesty

**King Edward's Hospital Fund for London**—(*continued*).
King George. We also realise the great position which His Royal Highness has already taken in British public work, and the Fund is to be congratulated that it can look forward to having his assistance in the future. At the same time, the hospitals are passing through difficulties even greater than those with which they were faced when the King's Fund was founded.

1919 AND 1897 COMPARED.

According to the estimate recently issued by the Honorary Secretaries of the Fund, the total expenditure of the London hospitals in 1919, taken together, was about £2,100,000. Towards this their income, including the grants from the three Central Funds, amounted to about £1,900,000; so that the deficit on the year's working was £200,000, or approximately 10 per cent. Now, if we turn back to the letter in which King Edward VII., then Prince of Wales, inaugurated the Fund in 1897, the year of the Diamond Jubilee of Her Majesty Queen Victoria, we find that the annual deficit of the London hospitals at that time was estimated at about £70,000, which, by an interesting coincidence, was also almost exactly 10 per cent., the total expenditure being then only £700,000. The amount distributed by the Fund in its first year was £57,000. Its subsequent success is reflected in the accounts before us to-day, which show that last year the Fund distributed £230,000, of which £116,000 was derived from investments.

ENERGY AND ORIGINALITY ESSENTIAL.

We know, of course, that the Voluntary Hospitals of London, with the aid of the Fund, survived the anxieties of 1897, and paid their way until the end of the war. Their expenditure during those twenty-three years increased threefold; but their income, including the Fund's grants, also increased at the same rate; and even in 1919 the deficit for the year was only the same percentage as in 1897. But we must not be too optimistic when we begin to think of the prospects of 1920 and the years to come. Of one thing we are certain: the present difficulties are not the result of any failure on the part of the hospitals, but arise solely from the general financial effects of the war. In order to surmount them very great energy will be needed and possibly even some new departure, requiring powers of imagination and originality equal to those shown by King Edward VII., when he inaugurated this Fund twenty-three years ago, if the hospitals are not to get into serious difficulties and to suffer in their great work. The result of King Edward's action then has been that the Fund now distributes a sum equal to one-tenth of the expenditure of the London hospitals, and distributes it so as to give most help to those hospitals which most need it, while at the same time encouraging them to assist themselves by efficiency and economy of management. I certainly feel that it is for us to see to it that when our President returns to his work in this chair he will find the Fund as active as ever in its task of assisting in the maintenance, extension, and improvement of the Voluntary Hospitals in London.

A DISTRIBUTION MEETING IN JULY.

Before I sit down there is one other announcement I ought to make. In an ordinary year there would not be another meeting of the Council at St. James's Palace until the Distribution meeting in December, when we all hope that His Royal Highness may be again in the chair. But this year there will be an extra meeting about the middle of July, for the purpose of deciding on the distribution of the sum of £250,000 which the Joint Finance Committee of the British Red Cross Society and the Order of St. John of Jerusalem has entrusted to the Fund for distribution in aid of schemes of extension or improvement which will directly or indirectly increase the facilities for the treatment of ex-sailors or ex-soldiers at the London Voluntary Hospitals. I am glad to see Sir Arthur Stanley here, and we shall look forward to the distribution of this money in the middle of July.

I now beg to move the adoption of the draft Report.

**The Governor of the Bank of England** (Mr. Norman), in seconding the resolution, said he desired to echo the words of the Chairman expressing the sense the members of the President's Powers Committee had of the great honour and privilege conferred upon them by his Royal Highness in delegating to them his powers, an honour and privilege which he especially felt as representing the Institution by reason of his connection with which he was present that day.

The adoption of the Report was then put and carried unanimously.

**The Chairman of the London County Council** (Mr. John W. Gilbert), in moving a vote of thanks to Lord Donoughmore for presiding, said he was sure that all present would agree that the statement made from the chair that day would prove a great advantage to the Fund.

The resolution was seconded by **Mr. John G. Griffiths**, who said that they were all very greatly indebted to the three members of the President's Powers Committee, Lord Donoughmore, Lord Finlay, and the Governor of the Bank of England, for undertaking the work. It was then put and carried unanimously.

**Lord Donoughmore** having briefly acknowledged the vote of thanks, the proceedings terminated.

